# Anthocyanin Bioactivity in Obesity and Diabetes: The Essential Role of Glucose Transporters in the Gut and Periphery

**DOI:** 10.3390/cells9112515

**Published:** 2020-11-20

**Authors:** Patrick Solverson

**Affiliations:** Department of Nutrition and Exercise Physiology, Elson S. Floyd College of Medicine, Washington State University, 665 N Riverpoint Blvd, Spokane, WA 99210, USA; patrick.solverson@wsu.edu

**Keywords:** obesity, type-2 diabetes, anthocyanins, GLUT2, GLUT4, AMPK, insulin sensitivity, mitochondria

## Abstract

Obesity and type-2 diabetes trends continue to worsen in the United States. Dietary anthocyanins (typically provided by berries and other fruits) are reported to have protective effects against both conditions using a variety of experimental research models including animal and human feeding studies. This review highlights studies that explore the biochemical pathways in both tissue and rodent models which could explain clinical improvements noted with anthocyanin consumption. First, the primary mode of intestinal absorption of anthocyanins is through both sGLT1 and GLUT2 glucose transporters. Stronger binding affinities may allow anthocyanins to be more inhibitive to glucose absorption compared to the reverse, where GLUT2 expression may also be affected. Genetic or chemical inhibition of sGLT1 or GLUT2 demonstrate their essential function in anthocyanin absorption across the enterocyte, where the former interacts with a greater variety of anthocyanins but the latter is the major transporter for specific anthocyanin-glycosides. Once absorbed, anthocyanins positively modulate GLUT4 density and function in both skeletal muscle and adipose tissues via the upregulation of AMPK and restoration of insulin sensitivity. Antioxidant properties and phosphodiesterase inhibition by anthocyanins promote both mitochondrial function and density which could be novel targets for dietary management of obesity and its complications.

## 1. Introduction

Obesity is a complex disease with worrisome trends. According to a recent analysis of the 2017–2018 National Health and Nutrition Examination Survey (NHANES), the overall prevalence of obesity (body mass index, BMI, >30) in the United States is 42.4% [1]. The rates of obesity are higher for minorities, females, lower socioeconomic status, and in midwestern and southern states. Further, adolescent obesity has quadrupled since the mid-1970s, and severe adult obesity (BMI >40) has doubled since the year 2000 [1,2,3]. Moreover, one projection estimates that 50% obesity prevalence in the United States will be reached by 2030 [4]. The health consequences of obesity are well documented and include cardiovascular disease, type-2 diabetes, several forms of cancer, and reduced mental health and quality of life [2]. A 2008 analysis of obesity’s economic impact estimated its comorbidities were the cause for 9% of annual medical spending, with the majority of expenditures dedicated toward pharmaceuticals for their management [5].

Insulin resistance and resultant type-2 diabetes are major complications of obesity, which further exacerbate health complications and damage to vital organs. In the United States, the prevalence of diabetes (fasting blood glucose above 126 mg/dL or hemoglobin A1c above 6.4%) in 2018 was 34.2 million people, or 10.5% of the population [6]. Of this estimate, 90–95% is type-2 (non-insulin dependent/adult-onset) diabetes. An additional one-third of US adults have prediabetes (fasting blood glucose between 100–125 mg/dL, or hemoglobin A1c between 5.7 and 6.4%). The greatest risk factors for type-2 diabetes are weight (89% of patients were overweight or obese), physical inactivity (38% of patients were inactive), and smoking (22% used tobacco) [6]. With poor dietary choices as a primary driver in obesity and type-2 diabetes, one area of nutritional sciences research is the bioactivity of functional foods, i.e., components within foods that may offer health benefits beyond classical forms of nutriture.

**Figure 1:**
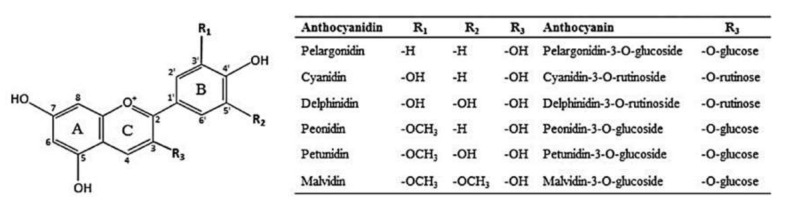
Structure of anthocyanidins commonly found in berry fruits and examples of corresponding anthocyanins with R_3_ glycosides. Reproduced with permission from [7].

One class of secondary plant metabolites to receive considerable attention as a candidate functional food ingredient are anthocyanins, a subclass of flavonoids responsible for the bright red to deep purple and blue pigmentation of commonly consumed fruits and vegetables (Figure 1) [7]. The best fruit sources of dietary anthocyanins in a typical American diet are from berries, grapes, and cherries, and vegetable sources include (red) cabbages, radishes, onions, eggplant, and black beans [8]. Given their wide variety and density (approximately 100–300 mg anthocyanins per 100 g fresh berries) it is notable that one USDA study using NHANES data estimates average daily consumption of anthocyanins in the United States is only 9 mg per day [9]. Presumably, anthocyanin intake would increase with better adherence to the dietary guidelines, or through the addition of functional food ingredients into commonly consumed food products.

**Figure 2:**
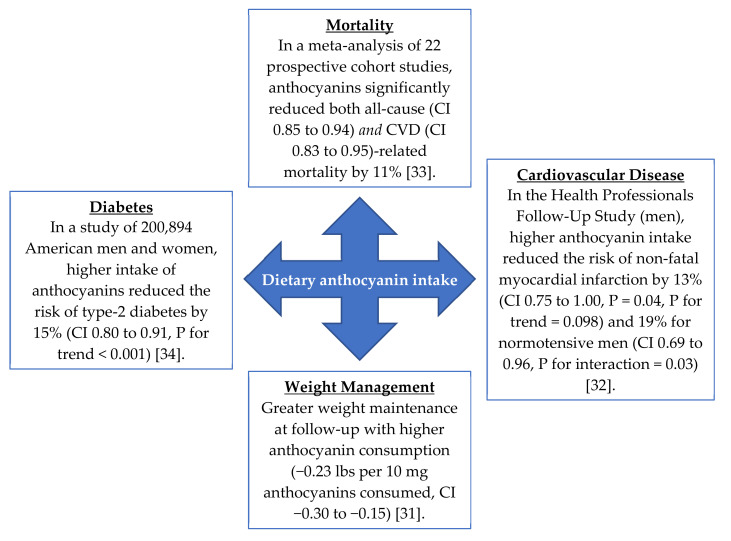
Anthocyanin consumption protects against age-related chronic diseases. CI; 95% confidence interval.

The bioactivity of berry anthocyanins has been described in rodent models of diet-induced and genetic forms of obesity and diabetes [10,11,12,13,14,15,16,17,18,19,20]. Further, positive health effects are also being reported in controlled clinical trials with berry interventions [21,22,23,24,25,26,27,28,29]. Improved insulin sensitivity is observed in overweight or obese subjects consuming anthocyanin-rich treatments [25,26,29]. One meta-analysis of five prospective cohort studies also supports the rodent and clinical evidence, where each 7.5 mg increment in daily anthocyanin consumption corresponded to an added 5% reduction in the risk of developing type-2 diabetes [30]. Pertinent epidemiological discoveries of anthocyanin consumption in human health are summarized in Figure 2 [31,32,33,34].

Translational research that explores the mechanistic action of anthocyanins is integral to a larger movement to identify food components and develop candidate crops, food products, and processing techniques that ultimately promote dietary patterns that offer protection against chronic disease [35,36]. How, mechanistically, can anthocyanins protect against the metabolic dysfunction associated with obesity and type-2 diabetes? The purpose of this literature review is to investigate the functional and regulatory molecular networks that respond positively to anthocyanin treatments, with an emphasis on intestinal and peripheral glucose transporters, and mitochondrial function. This review highlights research that demonstrates anthocyanins block sodium/glucose cotransporter 1 (sGLT1) and glucose transporter type 2 (GLUT2)-mediated intestinal glucose absorption, improve peripheral glucose transporter type 4 (GLUT4) function, and increase mitochondrial density.

## 2. Anthocyanins and Intestinal Glucose Transporters

The anti-glycemic effects of edible berries, reported in both human and animal experiments, warrant the elucidation of inhibitory effects of anthocyanins on glucose transport in intestinal cells. The Caco-2 cell line is a representative model system for the study of barrier function and cellular biology of the small intestine [37]. An anthocyanin extract from red grape skins, comprised of 90% malvidin glycosides, was tested in the Caco-2 model for both anthocyanin absorption as well as their influence on glucose absorption across the basolateral membrane [38]. Caco-2 cells, grown either in conventional conditions or pretreated with 200 µg/mL anthocyanins for 4 days, were tested for transport efficiency of anthocyanins out to 2 h from acute anthocyanin exposure with or without 1% ethanol. Transport efficiency increased across the 120 min, where both the addition of 1% ethanol, or 4-day anthocyanin pretreatment significantly increased the transport efficiency by 51% and 99%, respectively, compared to cells grown in control conditions. GLUT2 gene expression was significantly increased by approximately 50% with anthocyanin pretreatment, whereas GLUT5 and sGLT1 were not affected. Anthocyanin extract pretreatment significantly reduced Caco-2 glucose absorption by approximately 60%, comparable to treatment with purified malvidin-3-glucoside. The malvidin anthocyanidin (aglycone), did not interfere with glucose absorption, demonstrating the role of glycosylation for inhibition [38]. Degree of inhibition by different possible sugar moieties, for example, glucoside vs. galactoside vs. arabinoside (illustrated in Figure 1), would be useful to determine if glucose transport inhibition is also dependent on the type of anthocyanin glycosylation.

Polyphenol extracts from strawberry and apple juice were tested for their inhibitory effects on glucose transport into both Caco-2 cells and basolateral media [39]. Polyphenol interference on both sGLT1 and GLUT2 transport was tested by working in standard or sodium-free conditions. In addition to fruit juice extracts, constituent flavonoids, including pelargonidin-3-glucoside (P3G), were tested in purified forms in the same experimental conditions. IC_50_, reported as mg solid, was not different between strawberry and apple extracts for apical membrane transport of glucose. However, in both control or sodium-free conditions, apple extracts had about a five-fold greater inhibitory effect on basolateral glucose transport, indicating that both extracts inhibit glucose absorption into the cell, but apple polyphenols cross into Caco-2 cells more than strawberry polyphenols to further impede glucose transport. P3G could explain approximately 25% of the inhibitory action of the strawberry juice, whereas individual apple flavonoids, including quercetin-3-glucoside (Q3G), could explain 85% of its inhibitory action. Kinetic studies on the strawberry extract demonstrated reduced V_max_ and increased K_m_ with increasing dose of extract, indicating mixed-type inhibition on glucose uptake [39]. The apple extract yielded a stronger inhibitory effect compared to the strawberry juice, possibly because of the potency of Q3G [40].

In the same Caco-2 cellular model, an anthocyanin-rich mixed-berry extract was tested for influence on sodium-dependent and facilitative glucose transport [41]. The phenolic extract was 60% anthocyanins, and of that fraction 45% and 26% was cyanidin and delphinidin glycosides, respectively. Cells were treated in a dose-dependent manner up to 0.250% *w/v* extract, for up to 16 h, in culture media with or without sodium. GLUT2 and sGLT1 gene expression were significantly reduced by the extract after 12 h of pretreatment at higher (0.125% *w/v* or greater) doses. GLUT2, but not sGLT1 protein expression was also reduced by approximately 60% after chronic treatment. Total and facilitated glucose uptake was significantly reduced (approx. 40%, and 50%, respectively) after the acute treatment (15-min exposure with 0.125% *w*/*v*) with berry extract, but only facilitated transport was reduced after chronic extract exposure. Purified cyanidin aglycone, or two glycosylation states (glucoside or rutinoside), all significantly reduced the total and facilitated glucose uptake, which the authors note suggests steric interference instead of competitive inhibition, although this conclusion has been challenged with molecular docking studies, which suggest both steric and competitive inhibition [42,43]. Inhibitory effects were specific to the primary glucose transporters, as expression of GLUT5 (fructose transport) and GLUT1 (negligible role in intestinal glucose transport) were not significantly affected by extract treatment [41]. Therefore, anthocyanin interference with glucose transport is at least two-fold: by physically perturbing glucose absorption, as well as manipulating transporter expression in the intestinal cell.

The above work describes the interaction of anthocyanins with sGLT1 and GLUT2 to block glucose absorption. Another question is: “To what extent do anthocyanins rely on these two transporters for absorption across intestinal cells?” Cyanidin-3-glucoside (C3G) transport was tested in a Caco-2 transwell compartmental culture model with manipulation of transporter activity via both the use of inhibitors (phloridzin blocks sGLT1, and phloretin blocks GLUT2) or by transfecting the cells with small interfering RNA specific to one of the transporters [44]. Basolateral compartment C3G content was reduced after all four types of inhibition, demonstrating the involvement of both glucose transporters in the absorption of anthocyanins. Greatest C3G transport inhibition, up to a 50% reduction, resulted from blocking GLUT2 activity or expression, suggesting a greater role in C3G transport compared to sGLT1 [44]. The degree of inhibition to C3G transport with combination interference of both glucose transporters would help characterize possible compensatory mechanisms with other intestinal transporters thought to be involved with anthocyanin absorption, such as ATP-binding cassette and monocarboxylate transporters [42].

Anthocyanin absorption starts before the small intestine, as described in work with the MKN-28 gastric cell line, where red wine anthocyanin (RWA) transport was studied [45]. The motivation to work in a gastric cell model was to acknowledge the stomach’s role in the rapid appearance rate of anthocyanins in circulation after oral consumption [46,47,48]. A RWA extract, with malvidin-3-glucoside as the most prominent anthocyanin, was added (50 µg extract/mL media) to the apical side of a compartmental MKN-28 in vitro model for 180 min [45]. Manipulations included donor-side pH, anthocyanin preincubation, ethanol, and addition of glucose transporter inhibitor cytochalastin B to determine effects on anthocyanin transport efficiency. Compared to control conditions, preincubation with RWA (3 h incubation on two separate days of growing cells) significantly increased the transport efficiency by approximately 1.5% at 180 min. Increased apical pH (7.4) and addition of cytochalastin B both inhibited the transport efficiency, demonstrating significance of anthocyanin chemical structure (more stable at lower pH) as well as interaction with gastric glucose transporters. Moreover, increasing doses of D-glucose reduced RWA transport at all time-points measured, with D-glucose concentration ranging from 20–500 mM, indicating the reliance on glucose transporters for gastric absorption of anthocyanins. The MKN-28 model expresses GLUT1, GLUT3, and monocarboxylated transporter 1 (MCT1), but not GLUT2 or sGLT1.

In the same in vitro gastric model, pegylated gold nanoparticles were used to knockdown GLUT1 and 3 [49]. The findings confirmed GLUT1 and 3’s major role in gastric transport of anthocyanins, which could explain approximately 60% of anthocyanin transport to the basolateral compartment, but manipulation with Atropin and Verapamil indicate additional roles of organic cation transporter 1 and p-glycoprotein efflux transporters, respectively. Moreover, anthocyanin transport could not be completely blocked because of residual activity of constitutive GLUT1 and 3, confirmed through the addition of cytochalastin B. Molecular docking studies indicate roles of both the charge of the flavylium species (oxygen cation on the C-ring of the flavonoid structure) and glycosylation state in promoting passage across glucose transporters in gastric cells [49]. Beyond gastric GLUT1 and 3, absorption into the circulation is also possible via gastric bilitranslocase, where the presence of the anthocyanin’s sugar moiety promotes greater transit [50].

Anthocyanins from a bilberry extract have different rates of absorption, depending on both the aglycone and sugar moieties, as described in a combination pharmacokinetic (PK)/molecular modelling study [51]. Sprague Dawley rats were orally administered 100 mg/kg bilberry extract (Mirtoselect) under three conditions: fed, fasted, or fasted in combination with 1 g/kg glucose, in order to determine the effects of glucose on anthocyanin absorption. Blood samples were collected for 24 h. The bilberry extract contained 36% anthocyanins and 15 different forms were identified, including cyanidin, peonidin, delphinidin, petunidin, and malvidin, each with arabinose, galactose, and glucose moieties. PK revealed a dramatic effect of fed state on anthocyanin absorption, where fasted animals increased anthocyanin absorption into the plasma compared to fed by over 7-fold. In contrast, there was no interference of the presence of glucose on total absorption of bilberry anthocyanins. However, it did shift the T_Max_, the time it takes to reach maximum plasma anthocyanin concentration, from 15 min to 30 min after gavage, indicating an influence on kinetics, but not total amount absorbed, as AUC was not different [51]. Conversely, as mentioned above [38,39,41] and elsewhere [7,52] this interaction of glucose and anthocyanins in the gut does affect the total glucose absorption. Baron et al. therefore conclude that the binding constant for the GLUT transporters between anthocyanins and glucose should be in contrast, as evidence suggests anthocyanins block glucose, but not vice versa [51]. Both anthocyanidin and glycoside moieties influenced the relative abundance in plasma appearance when compared to abundance in the bilberry extract. Interestingly, cyanidin-3-glucoside and -galactoside, and peonidin-3-galactoside had greater relative abundance in plasma compared to extract, and the majority of arabinoside-containing anthocyanins were poorly absorbed. Docking studies demonstrated that there is greater interaction with several anthocyanin and glycoside transition states with the sGLT1 gut transporter compared to GLUT2. This is explained by the charge of the residues in the binding cavities of the transporters, where GLUT2 preferentially interacts with the neutral tautomer, whereas sGLT1 could interact with the majority of forms studied [51]. Similarly designed experiments, including PK of individual anthocyanins and molecular docking models, would be informative in human studies.

Anthocyanin transport studies were performed with Ussing chambers and mouse jejunal tissues [53]. A boysenberry extract was the source of C3G. C3G absorption into tissue lysates (5 µM C3G in Ringer’s solution) was tested for inhibition by increasing the concentrations of glucose (10–40 mM), phloridzin (50–200 µM), or the presence of Q3G (50 µM). C3G absorption, measured as disappearance from Ringer’s solution, was not significantly inhibited by the presence of glucose or the sGLT1 inhibitor phloridzin. This observation supports the findings of others described above: C3G interferes with glucose absorption, not vice versa, and GLUT2 acts as its major transporter. Interestingly, Q3G significantly interfered with C3G absorption, reducing its percent disappearance from Ringer’s solution by 74%, indicating competition for the same transport mechanisms [53]. It is notable that a ten-fold higher concentration of Q3G was used relative to C3G. Walton et al. noted that others [54] have demonstrated that the presence of glucose interferes with Q3G absorption, and therefore conclude that Q3G relies on sGLT1, whereas C3G does not. These findings cast doubt on the bioactivity of C3G as it shows Q3G will greatly inhibit its absorption. However, the other work discussed in this review where crude fruit extracts were used typically contain Q3G, and C3G’s absorption was retained. Further, blocking C3G absorption does not equate to ablation of bioactivity as the microbial metabolites of C3G possess bioactivity and therefore must also be considered (discussed later). The other work on C3G transport efficiency described above noted impediment of C3G transport in the presence of 20–500 mM glucose in gastric cells [45]. The physiological significance of treatment arms with very high (>50 mM) glucose concentrations can be challenged, as the physiological range of luminal glucose is 0.2 to 48 mM [55]. However, higher concentrations are typically achieved in mechanistic studies harnessing direct infusion or perfusion techniques, and are useful to identify the functional, i.e., hormonal regulation, translocation, and kinetic aspects of sGLT1 and GLUT2 [56,57,58]. Conversely, a human study observed a 20% reduction in urinary anthocyanins when 11 g of an elderberry extract was consumed with 30 g sucrose, suggesting interference of anthocyanin transport by glucose conceivably because of the competition for the same glucose transporters in the gut [59]. Collectively, these studies suggest flavonols, anthocyanins, and glucose all impede one another’s absorption in the gut. However, the aforementioned work may suggest a more dramatic effect of anthocyanins on glucose transport, whereas fed state and presence of other flavonoids may be more detrimental to the successful passage of anthocyanins across intestinal glucose transporters.

## 3. Anthocyanin Action on Regulatory Pathways Involving GLUT4 Function in Cellular and Rodent Models of Metabolic Dysfunction

Beyond blocking intestinal glucose absorption, anthocyanins modulate cellular metabolism in peripheral tissues. The in vivo and in vitro work discussed below presents additional potential for anthocyanins to ameliorate hyperglycemia, and other metabolic complications of a poor diet and sedentary lifestyle. The studies summarized below highlight anthocyanin action, which includes restoring insulin signaling, GLUT4 expression and function, and reversing mitochondrial stress.

In an early report of the influence of anthocyanins on this network, type-2 diabetic (KK-A^y^) mice were fed control or control + 0.2% C3G diet for 5 weeks to determine the clinical and biochemical outcomes [60]. Special attention was given to the effect of C3G on retinol binding protein 4 (RBP4), which is elevated in rodent and human obesity and type-2 diabetes [61]. C3G dose was based on earlier work [62] where food intake was not affected. C3G significantly reduced blood glucose and improved insulin sensitivity independent of adiponectin. Fasting glucose was significantly reduced by 22% with C3G treatment at 5 weeks. After insulin injection, the C3G group had 0.83, 0.67, and 0.81-fold lower blood glucose at 30, 60, and 120-min timepoints, respectively, compared to control. C3G increased gene expression of GLUT4 by 2.4-fold in white adipose tissue (WAT), which other reports show is reduced in type-2 diabetes [61,63,64]. Moreover, the authors found increased GLUT4 protein expression in both whole cell lysate (2.6-fold increase) and the plasma membrane (3.1-fold increase) of WAT with C3G treatment. C3G reduced WAT expression of retinol-binding protein 4 (RBP4) by 53%. RBP4 gene expression was unchanged in the liver, and its serum concentrations were reduced by 47% with C3G. C3G treatment also reduced tumor necrosis factor-alpha (TNF-α) and monocyte chemoattractant protein-1 (MCP-1) expression in the WAT by 76% and 47%, respectively. MCP-1 has been shown to play a central role in insulin resistance in WAT, where its administration can block the rescue of GLUT4 [65]. Collectively, the downregulation of RBP4 in both WAT and circulation, as well as the WAT-specific reduction in cytokines could help explain the increase in insulin sensitivity. Sasaki et al. note their observed changes in WAT and falling RBP4 trigger a reduction in glucose-6-phosphatase, thereby reducing hepatic glucose output, aiding euglycemia [60]. In their proposed model of C3G action in WAT, the cascade starts with the upregulation of adenosine monophosphate-activated protein kinase (AMPK). It is combined with C3G’s antioxidant and anti-inflammatory activity, which leads to positive downstream effects on GLUT4 expression and translocation in WAT, which reduces RBP4 expression. This ultimately restores metabolic function in liver and skeletal muscle to improve insulin sensitivity and glycemia.

Other dietary sources of anthocyanins have positive effects on GLUT4. Streptozotocin-induced diabetic rats were treated with anthocyanins from black soybean seedcoats to test for molecular and clinical improvements [66]. The anthocyanin treatment was compared to glibenclamide, a reference sulfonylurea for the pharmaceutical management of type-2 diabetes, which promotes hypoglycemia via the stimulation of insulin secretion. The anthocyanin-rich black soybean extract (BSE, containing 72% C3G) was administered orally (50 mg/kg/day) via liquid gavage for 30 days. BSE action in diabetic rats was numerous, including anti-apoptotic effects in the pancreas (increasing serum insulin as effectively as glibenclamide), increased antioxidant activity in serum, and activation of insulin receptor-β subunit in both skeletal and cardiac muscle. GLUT4 protein expression was increased between 3 and 4-fold compared to control diabetic rats in both tissues, which Nizamutdinova et al. suggest is caused by insulin receptor-β activation. BSE also led to significant improvements in clinical and cardiac measures. In all measures, BSE improved STZ-induced outcomes at or better than glibenclamide treatment, including superior modulation of GLUT4 protein expression in both tissues. The multitude of effects attributable to BSE, including both promotion of insulin secretion from the pancreas as well as acting as an insulin mimetic itself, likely contributes to its potency in remediating the complications of diabetes.

Another berry study fed type-2 diabetic KK-A^y^ mice a control diet ± 1% bilberry anthocyanin extract (BBE) for 5 weeks to determine the clinical and biochemical effects [67]. 1% diet supplementation with BBE did not reduce food intake. The extract was a heterogenous mixture of anthocyanin glycosides with major forms including delphinidin, petunidin, malvidin, and cyanidin backbones attached to a glucoside, arabinoside, or galactoside moiety. Starting at week 3, serum glucose was approximately 30% lower in mice fed the BBE, despite no difference in serum insulin by week 5. A 2-h insulin tolerance test at week 5 confirmed an increase in insulin sensitivity with BBE, where 3 of the 4 timed measurements (30, 90, and 120 min) on serum glucose were significantly lower in the BBE-fed group compared to control after insulin injection. Like Sasaki et al.’s findings [60], RBP4 gene expression was downregulated in WAT in mice receiving BBE, however, RBP4 protein expression in both serum and WAT was not different. Increased activated (phosphorylated) AMPK promoted 2.1 and 2.3-fold increases in GLUT4 protein expression in WAT and skeletal muscle, respectively, with BBE treatment. The responses were independent of insulin receptor substrate-1 (IRS-1) and protein kinase B (Akt), which were unchanged. AMPK activation in the liver (about 1.3-fold higher than control) also promoted down regulation of gluconeogenic enzymes and a reduction in hepatic glucose output following a pyruvate tolerance test. Serum triglycerides and cholesterol were significantly decreased by a respective 31% and 15% with BBE, which can be explained by activated hepatic AMPK upregulating gene expression of peroxisome proliferator-activated receptor-alpha (PPAR-α), acetyl-CoA oxidase, and carnitine palmitoyl transferase 1-alpha (CPT1-α), and downregulating acetyl-CoA carboxylase. Takikawa et al. note that anthocyanin bioactivity from bilberries is by way of insulin-independent mechanisms (AMPK activation), across several peripheral organs, to improve the clinical complications of type-2 diabetes, including hyperglycemia, dyslipidemia, and insulin resistance [67]. They also note the same AMPK pathway is activated in type-2 diabetics via exercise regimens [68]. No changes in adiponectin were observed. One limitation of this study is the high dose of BBE fed to mice. Using the FDA’s human equivalent dose (HED) guidance [69,70], their dose would correspond to approximately 115 mg bilberry anthocyanins/kg body weight. A 60 kg human would need to consume 6.9 g of bilberry anthocyanins, or 18.4 g of BBE, which would not be achievable through normal fruit consumption. This disconnect in achievable dose is a major limitation for some of the translational work surrounding the bioactivity of anthocyanins if one were to consider the levels provided by a typical human diet. However, these equivalent doses in humans are possible through dietary supplements, therefore their potential bioactivity and therapeutic benefit should not be dismissed entirely [70].

Black soybean seed coats are an abundant source of cyanidin, petunidin, and delphinidin, and they are commonly consumed as a tea in Japan. Its extract was studied in both db/db (type-2 diabetic) mice as well as in 3T3-L1 adipocytes to determine the effects on clinical, morphological, and molecular variables [71]. Culture experiments also included a purified form of C3G for comparison. Lean and db/db mice were fed a control diet and treated orally with either water (control) or 30 mg/kg BSE daily for 30 days. Major findings from the animal study included decreased body and white adipose depot weights, however the BSE treatment group consumed significantly less diet. In BSE-treated 3T3-L1 adipocytes, histological examination showed they were smaller in size compared to control. In adipocytes treated with 20 and 100 µM pure C3G, major findings included over four-fold increase in adiponectin secretion, and a 70% reduction in TNF-α production. Major changes in gene expression include upregulation of CCAAT/enhancer-binding protein alpha (C/EBPα), insulin receptor and Akt activation, and over 2-fold increase in GLUT4, which corresponded to a 1.8-fold increase in glucose uptake. Moreover, C2C12 skeletal muscle cells grown with media conditioned by 3T3-L1 adipocytes treated with 100 µM C3G showed significant 2, 1.4, and 2.4-fold increases in peroxisome proliferator-activated receptor gamma coactivator 1-alpha (PGC-1α), sirtuin 1 (SIRT1), and uncoupling protein 3 (UCP3) gene expression, respectively. These observations would suggest increases in mitochondrial biogenesis and capacity for oxidative phosphorylation. The C2C12 model effects were thought to be caused by the increased secretion of adiponectin by the C3G-treated adipocytes. These findings contrast with the earlier study, which suggested positive changes in KK-A^y^ mice were independent of adiponectin [67]. These contrasting findings may suggest that similar clinical improvements can be reached through discordant mechanistic activity, threshold doses, or transient increases in adiponectin. Differences in anthocyanin dose and/or the presence of other bioactive polyphenols in both extracts could help explain why adiponectin response is not a prerequisite to clinical improvement.

A follow-up study pursued the potential for white-to-brown induction, or “beige” phenotypic switch, by C3G treatment in differentiating 3T3-L1 adipocytes [72]. The conversion of white to brown adipocytes has gained attention as a possible adjuvant to obesity treatment; brown adipose tissue (BAT) has higher mitochondrial content and oxidative capacity because of its higher expression of UCPs, which causes less efficient capture of energy via loss through thermogenesis [73,74,75,76]. During cellular differentiation, 3T3-L1 adipocytes were grown with increasing doses of purified C3G: 0 µM (control), 50 µM, and 100 µM. Major findings include morphological changes of 3T3-L1 cells to characteristics representative of BAT, including presentation of multilocular lipid droplets, which was attributed to increased cellular differentiation. Increased cellular differentiation was supported by up to a 2.4-fold increase in fatty acid binding protein 4 (FABP4) gene expression. The C3G-treated 3T3-L1s also presented a 20% increase in mitochondrial content, 40% increase in activated AMPK protein expression, up to two-fold increases in mitochondrial genes, including UCP1 and UCP2, as well as increased protein expression of UCP1 and PGC-1α compared to control. The increased mitochondrial density is due to the upregulation of mitochondrial transcription factor A (TFAM) via the activation of AMPK, which is activated by falling ATP levels in response to greater uncoupling by UCPs. Genetic markers of BAT, TBX1, and CITED1, were monitored throughout differentiation, and both levels of C3G significantly increased their expression from day 3 to day 7 compared to control. Matsukawa et al. hypothesize that the phenotypic switch is caused by the increase in cAMP through C3G’s inhibition of phosphodiesterases, which upregulates C/EBPβ, thereby promoting the beige cell phenotype via activation of PGC-1α and PPARγ [72].

Purified C3G’s effects on molecular pathways in adipose tissue have also been investigated in vivo. Db/db mice were fed C3G dissolved in drinking water (1 mg/mL; HED = 22 mg/kg, or 1320 mg of C3G for a 60 kg adult) or control for 16 weeks. Starting at 3 weeks, mice fed C3G gained less weight than their control counterparts and were approximately 12% lighter by week 16. Liver, subcutaneous white adipose tissue (sWAT), and epididymal white adipose tissue (eWAT) also weighed significantly less in the C3G group. Similarly, BAT, sWAT, and eWAT lipid droplet diameter was smaller. In the light cycle, when mice are inactive, C3G-treated mice consumed about 30% more oxygen (higher energy expenditure) despite no difference in energy intake or physical activity. Glucose AUC from both a glucose or insulin challenge was improved after C3G treatment, in addition to a superior lipid profile and liver histology. BAT exhibited greater activity, i.e., higher body temperature after cold stimulation, and increased uptake of 18F-FDG in a PET-CT scan. Molecular pathways related to mitochondrial biogenesis (PGC1-α, nuclear response factor (NRF)1/2, and TFAM) and activity (UCP1 and CPT1-α) were all significantly elevated in BAT with C3G treatment. These same markers, as well as indicators of a “beige” or “brite” effect in sWAT (transmembrane protein 26, CD137, and T-box protein 1) were also upregulated. SIRT1, the histone deacetylase which garnered considerable attention for its activation by the stilbene resveratrol [77], was also increased in the adipose tissue, which was suggested to be responsible for the activation of PGC1-α and subsequent stimulation of TFAM and mitochondrial biogenesis.

Recent work on anthocyanin bioactivity in adipocyte culture stands in stark contrast to the studies described above [78]. Pelargonidin, a common anthocyanin in strawberries and grapes, was studied in its purified form in the 3T3-L1 adipocyte model to determine its influence on growth and differentiation. The cells were treated with increasing doses of pelargonidin in the second half of the differentiation period (2 days). Pelargonidin doses up to 200 µM were reported to have no negative effect on cell viability, therefore observed effects of treatment could not be explained by cell toxicity. Main results included reduced lipid staining, TG content, and glucose absorption. Compared to control cells, the highest pelargonidin dose tested, 20 µM, reduced lipid accumulation by 48% and increased extracellular media concentration of glucose over seven-fold. Mechanistically, with the same dose, PPAR-γ protein expression was reduced to 21% of control, and other affected targets include HMGCR, lipoprotein lipase, FABP4, and an approximate 80% reduction in GLUT4 protein expression [78]. Methodological differences in culture experimentation could explain the divergent findings of this study, as other work described above with C3G has good agreement across cell culture and rodent studies, whereas the pelargonidin action described here is not supported by others.

Anthocyanin metabolites also show GLUT4-stimulating activity. A microbially derived circulating metabolite of C3G and other anthocyanins, protocatechuic acid (PCA), has demonstrated antioxidant, anti-atherosclerotic, and PPARγ-induced insulin-mimetic activities [79,80]. Given this potential, PCA was studied for its ability to augment insulin sensitivity in primary adipocytes from human donors after treatment with oxidized LDL (oxLDL) [81]. Primary adipocytes were pretreated with 25 µM PCA for 2 h before 4 or 18 h treatments with 100 mg/dL oxLDL. The insulin signaling pathway, glucose uptake, and GLUT4 expression were measured for influence of PCA treatment. IRS-1 protein expression, reduced to approximately 50% with oxLDL treatment, was rescued to control levels with PCA preincubation. Further, PCA normalized the increase in serine-phosphorylated (inactivated) IRS-1 caused by oxLDL. In non-oxLDL stressed cells, PCA interacted with (activated) each component of the IRS-1/phosphatidylinositol 3-kinase (PI3K)/Akt insulin-signaling pathway to a degree not different from insulin (control) stimulation in serum-starved primary adipocytes, where activation (phosphorylation) of AMPK was also not different between PCA and insulin treatments. The observed activation of AMPK was attributed to PCA’s ability to stimulate adiponectin [80]. Experiments with IRS-1 and PI3K inhibitors showed that PCA’s positive effect on glucose uptake and GLUT4 translocation is specific to IRS-1 pathway activation, as use of either inhibitor abrogated the PCA-induced enhancement of GLUT4. Given the positive molecular effects of PCA, Scazzocchio et al. note that it reaches a C_max_ of 10 µM in human plasma, attributable to both intestinal absorption of anthocyanins as well their breakdown and subsequent PCA production in the large intestine [82,83]. However, the microbial degradation of anthocyanins and bioactivity of their metabolites is very complex and cannot be simplified to a single metabolite; the reader is referred to a comprehensive review on the topic [84]. Regardless, these findings on the molecular effects of PCA are supported by in vivo work that suggest the studied concentrations may be physiologically relevant.

In obese Zucker rats (OZR), 8% wild blueberry (WB) powder improved inflammation, lipid metabolism, and endothelial function after 8 weeks of treatment [85,86,87]. With an emphasis on glucose metabolism, a follow-up study fed OZR the same 8% WB diet for 8 weeks and measured fasting glucose, insulin, glycated hemoglobin, RBP4, and resistin, as well as gene expression of GLUT4, resistin, and RBP4 in both liver and adipose tissues [88]. An 8% WB diet provided 0.12% anthocyanins, primarily in the form of malvidin and peonidin glycosides. Such a dose, using the standard body surface area equation [69,70], is equivalent to a human consuming two cups of blueberries per day. When fed WB, OZR had a 20% reduction in glycated hemoglobin compared to control, but fasting glucose and insulin were not different. However, there was a significant 22% and 27% reduction in plasma RBP4 and resistin, respectively, in OZRs fed WB compared to control. There was no effect on GLUT4 expression in liver or adipose tissues. However, resistin gene expression was significantly reduced in the liver but not adipose tissue of the WB-fed animals. Conversely, RBP4 gene expression was significantly reduced by approximately 90% in the adipose tissue of WB-fed OZR but not in the liver. Increased glucose tolerance with WB treatment, evidenced by improved glycated hemoglobin, was thought to be due to significantly lower circulating resistin, which is responsible for interrupting the normal insulin signaling cascade in peripheral tissues. The anti-inflammatory effect of WB, combined with upregulation of PPARγ, may explain the reduction of RBP4 in both plasma and adipose tissue, further alleviating insulin resistance, but the authors suggest gene expression remained constant in the liver possibly because of RBP4’s role in Vitamin A metabolism [88]. The lack of an effect on adipose GLUT4 and serum glucose and insulin may suggest a threshold dose-effect was not reached with the level of anthocyanins supplemented in this rodent diet or because of the variable rate of intestinal absorption among different anthocyanins described earlier.

Another low(er) anthocyanin dose rodent study fed db/db mice one of three diets for six weeks: control, 0.005% *w*/*w* rosiglitazone (an oral anti-diabetic agent), or 0.5% *w*/*w* mulberry extract (ME) [89]. The ME contained both C3G and cyanidin-3-rutinoside (C3R), as determined by HPLC. Fasting blood glucose was lowest in rosiglitazone-treated db/db mice starting at week 2 and remained approximately 4-fold lower than control at week 6. ME-treated mice had lower fasting glucose compared to control starting at week 3 and was approximately 1.5-fold lower by week 6. Similarly, glycated hemoglobin, fasting insulin, and homeostasis model assessment of insulin resistance (HOMA-IR) were all significantly lowered by 30%, 30%, and 57%, respectively, with ME treatment compared to control, but not to the same degree as rosiglitazone, which yielded the most dramatic improvements. 2-h intraperitoneal glucose and insulin tolerance tests resulted in similar findings; ME treatment lead to improvements in glucose and insulin tolerance compared to control mice, and the improvement in plasma insulin was not different between ME and rosiglitazone-treated groups. Plasma membrane and total GLUT4, activated AMPK, and activated and total Akt substrate (AS160) were all significantly improved in the skeletal muscle of mice treated with ME compared to control by approximately 1.8, 1.2, 1.6, 2.1, and 1.25-fold, respectively. Again, all measures, except total GLUT4, were not increased to the same extent as rosiglitazone treatment. Similar findings were true with hepatic expression of activated AMPK and gene expression of G6Pase and PEPCK, despite similar increases in liver glycogen of rosiglitazone or ME-treated mice compared to control. Despite superior performance of rosiglitazone compared to ME in most measures, it is noteworthy that rosiglitazone-treated mice were 22% heavier than control or ME-treated mice, despite no difference in food intake, and saw a significant increase in adipose tissue weight, a common undesirable side-effect of its treatment [89]. ME treatment yielded increases in insulin sensitivity because of increased uptake of blood glucose by skeletal muscle tissue as well as reduced hepatic gluconeogenesis and glucose output.

To further characterize the glucoregulatory effects of C3R, 3T3-L1 adipocytes were treated with a range of purified C3R, from 5–50 µM, for 2 h before measuring glucose uptake [90]. Increased glucose uptake was noted starting with 10 µM C3R (17% increase) and increased up to 69% with the 50 µM compared to untreated cells. 50 µM C3R significantly increased the activation of IRS-1, Akt, and protein expression of PI3K compared to control by 1.39, 1.37, and 1.56-fold, respectively. The increased Akt and PI3K with C3R were comparable to stimulation with 100 nM insulin. Moreover, the activation was not due to the changes in AMPK expression. However, the use of inhibitors wortmannin and compound C (PI3K and AMPK/Akt inhibitors, respectively) demonstrated that C3R’s influence on GLUT4 translocation to the plasma membrane, and subsequent increase in glucose uptake, is dependent on AMPK activity despite not affecting expression directly. Therefore, the author’s proposed site of activation by C3R and subsequent modulation of the insulin signaling cascade is at the level of tyrosine phosphorylation of IRS-1 [90].

Several anthocyanins were extensively studied for effects on the molecular mechanisms of 3T3-L1 adipocyte metabolism [91]. Anthocyanin treatments included a purple corn extract (PCE) or purified forms of cyanidin, pelargonidin, and peodin-3-glycosides (C3G, Pr3G, and P3G, respectively) in doses ranging from 15 to 125 µM. According to a lipid accumulation assay, a 50% reduction in adipocyte differentiation is achieved with the PCE that provided 50 µM C3G-equivalents. Of the purified anthocyanins, C3G had the greatest inhibitory effect on PPAR-γ transcriptional activity with an IC_50_ of 11 µM. PCE and purified anthocyanins had divergent effects on lipolysis and fatty acid synthesis, where PCE had greatest inhibition on triglyceride content in basal cells, and the reverse was true for purified anthocyanins in adipocytes stimulated with high-glucose media for 4 h to model post-prandial conditions. Enzymatic assays and molecular docking studies confirmed the inhibitory effect of the anthocyanins on fatty acid synthesis as well as promotion of lipolysis in adipocytes. An insulin-resistant adipocyte model demonstrated the antioxidant, anti-inflammatory, and insulin-sensitizing activities of both the PCE as well as the purified anthocyanins. Notably, PCE increased the total GLUT4 protein expression and membrane translocation compared to TNF-α-treated control adipocytes by 2.9 and 7.6-fold, respectively. Similarly, purified C3G demonstrated the same improvements by 1.9 and 6.4-fold compared to control. The PCE had greatest protective effects, followed by C3G, suggesting that the PCE yields a synergistic effect of its anthocyanin components, and that C3G likely elicits the greatest bioactivity among them.

Grape skins, also a rich source of anthocyanins, modulate AMPK and GLUT4 in vivo. A recent study prepared a grape skin extract (GSE), high in peonidin, petunidin, and malvidin glucosides, and fed C57BL/6 mice a 60% high fat diet (HFD) with or without an additional oral gavage of 200 mg/kg/day GSE for 12 weeks [92]. HFD-fed mice administered GSE were protected from the characteristic weight gain attributable to high-fat feeding starting at day 40 and were not different from control (normal fat) diet animals through week 12. The GSE improved fat pad weights and morphology as well as clinical measures of insulin sensitivity and dyslipidemia. In-line with improved insulin sensitivity, the GSE treatment improved or normalized components of the IR/IRS/PI3K/Akt pathway. In the skeletal tissue of mice fed the HFD, GSE treatment normalized activated AMPK and GLUT4 protein expression to control diet levels. Moreover, GSE increased GLUT4 expression by about 65% compared to the HFD group in the adipose tissue. GSE improved adipokines in both adipose tissue and plasma, as well as prevented oxidative damage in plasma, adipose, and skeletal muscle. The findings are concordant with the other experiments administering GSE in diabetic rat models and offspring of HFD-fed dams [93,94]. Collectively, these studies provide further evidence that improved clinical complications of diet-induced obesity are possible by administration of anthocyanin-rich extracts. These extracts can cause the abrogation of inflammation and oxidative stress as well as a restoration of insulin sensitivity, which is dependent on a return to normal GLUT4 expression and function.

Similar in vivo work has been performed with blackcurrants, a dark colored berry produced mainly in Europe and New Zealand. A rich source of delphinidin-3-rutinoside (D3R), blackcurrants were studied for their antidiabetic effects in the KK-A^y^ type-2 diabetic mouse model [95]. A D3R-rich source was desired as earlier phenotyping highlighted its ability to stimulate GLP-1 in cells and in rats given intraperitoneal glucose injection [96,97]. 5-week-old KK-A^y^ mice were fed either a standard chow diet or diet supplemented with 1.1% blackcurrant extract (BCE) for 7 weeks. The BCE was composed of 45% anthocyanins (19.3% of which as D3R) and 82% total polyphenols. Despite no changes in body weight, food intake, or plasma insulin concentrations, weekly fasting serum glucose was lower in mice fed the BCE starting at week 2 and remained about 20% lower than control mice through week 7. BCE-fed mice also had significantly lower serum glucose (lower at 15 and 60 min) when administered an OGTT. Main molecular mechanisms altered by the BCE include significant 50% increases in ileal proprotein convertase 1 (PC1/3) gene and protein expression. PC1/3 is the enzyme responsible for converting proglucagon into GLP-1, an incretin hormone produced by the small intestine responsible for stimulating insulin secretion. Plasma GLP-1 concentration was about 30% higher in the BCE-fed mice. Activated AMPK was also elevated by BCE treatment in both the skeletal muscle and liver, where there was a corresponding 40% increase in plasma membrane GLUT4 in skeletal muscle, however PEPCK and G6Pase gene expression were unaffected by the increase in activated AMPK in the liver. The stimulation of both GLP-1 and AMPK are reasons for the improvements in the T2D mouse, but Izuka et al. are uncertain of the mechanism behind the stimulation in PC1/3 expression by BCE. Their earlier work suggests it is the parent D3R exerting bioactivity to stimulate postprandial GLP-1 secretion [97].

## 4. Anthocyanins Block Reactive Oxygen Species and Protect Mitochondrial Function

D3R has been tested for protective effects against oxLDL-induced mitochondrial dysfunction and apoptosis in human umbilical vein endothelial cells (HUVECs) including the potential role of sGLT1. In HUVECs exposed to 100 µg/mL oxLDL for 12 h, a 2-h pretreatment with 10–200 µM D3G increased cell viability and reduced the percentage of apoptotic cells from 60% (control oxLDL exposure) down to 20% (10 µM D3G pretreatment) or 10% (50–100 µM D3G pretreatments). Further, ROS and superoxide generation, mitochondrial membrane potential, and mitochondrial permeability transition pore opening were all improved with 10–100 µM D3G pretreatments compared to control oxLDL-treated cells. D3R uptake into HUVECS could be blocked via 0.25- or 0.50-mM phlorizin, in sodium-free conditions, with 10–100 µM glucose, or with siRNA specific to sGLT1. Moreover, the dose-dependent protection offered by 10 and 100 µM D3G on cytochrome C release, Bax/Bcl-2 ratio, and caspase 3, all indicators of mitochondrial dysfunction, could be abrogated with sGLT1 siRNA or 0.25 mM phlorizin. Therefore, the mitochondrial protection offered by D3G in oxLDL-induced stress and apoptotic endothelial cells operates through a pathway that is dependent on D3G cell entry via sGLT1. Interestingly, mice given IV injections of D3R showed higher concentrations in their thoracic endothelial cells compared to plasma, suggesting the ability of anthocyanins to concentrate in tissues despite low levels in the circulation. Elucidating the mechanisms by which D3R protects mitochondrial function, after entry into the cell via sGLT1, are warranted. Moreover, the dependence of D3R cellular entry with sGLT1, as well as mechanisms of inhibition and enhancement, could also be explored in the heart and kidneys.

The delphinidin (delph) aglycone has also been studied for its antioxidant properties in other tissue models of diabetes [98]. In a cellular model of diabetic nephropathy, 24 h incubation of mesangial (glomerular) cells with 25 mM glucose induces cellular proliferation and excess extracellular matrix, which are triggered by mitochondrial stress stemming from NADPH oxidase 1 (NOX1) activation and ROS generation. When cells were pretreated for 1 h with 50 µM delph before 24 h glucose treatment, collagen formation and cellular proliferation were abrogated. ROS generation and NOX1 activation were also blocked by delph, which showed a stronger protective effect relative to cyanidin and malvidin aglycones. Activation of extracellular signal-regulated kinase (ERK)1/2 by ROS increases cellular proliferation and collagen synthesis via activation of transforming growth factor beta (TGF-β), both of which were shown to be normalized with delph treatment. The findings demonstrate how delph blocks collagen synthesis and proliferative pathways at the level of ROS, which is likely due to its antioxidant activity. However, the work discussed above suggests other possible mechanisms by which delph could impede the cellular stress of a high glucose environment, such as blocking glucose transport into the cell and/or promoting mitochondrial function, therefore it would be informative to build upon this work to see how precisely delph is preventing the glucose-induced rise in ROS beyond its antioxidant properties.

**Figure 3:**
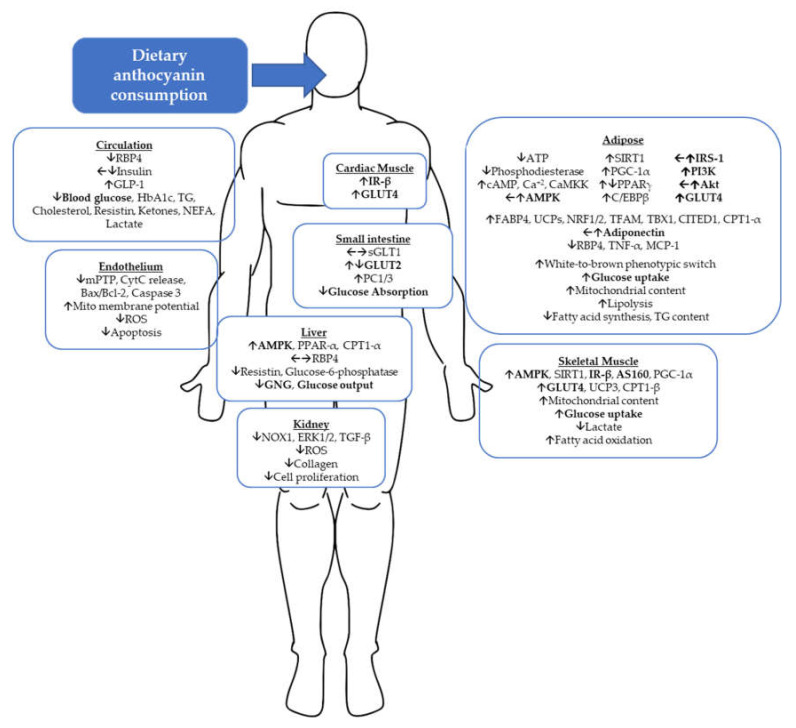
Summary of anthocyanin action on molecular targets and corresponding functional changes across tissues. Up-arrow; increased expression, activation, function, or concentration, sideways-arrow; no change, and down-arrow; a decrease. Multiple arrows on the same target indicate different findings across discussed studies. Bolded targets are directly related to glucose transporters and glucose homeostasis. RBP4; retinol binding protein 4, GLP-1; glucagon-like peptide 1, HbA1c; hemoglobin A1C, TG; triglycerides, NEFA; non-esterified fatty acids, mPTP; mitochondrial permeability transition pore, CytC; cytochrome C, ROS; reactive oxygen species, IR-β; insulin receptor-beta, GLUT4; glucose transporter type 4, sGLT1; sodium/glucose cotransporter 1, GLUT2; glucose transporter type 2, PC1/3; proprotein convertase 1/3, AMPK; adenosine monophosphate activated protein kinase, PPAR-α; peroxisome proliferator-activated receptor, CPT1-α; carnitine palmitoyl transferase 1, GNG; gluconeogenesis, NOX1; NADPH oxidase 1, ERK1/2; extracellular signal-regulated kinase 1/2, TGF; transforming growth factor, cAMP; cyclic adenosine monophosphate, CaMKK; Ca^2+^/calmodulin-dependent protein kinase kinase, SIRT1; sirtuin 1, PGC-1α; peroxisome proliferator-activated receptor gamma coactivator 1, C/EBP; CCAAT/enhancer-binding protein, IRS-1; insulin receptor substrate 1, PI3K; phosphatidylinositol 3-kinase, Akt; protein kinase B, FABP4; fatty acid binding protein 4, UCP; uncoupling protein, NRF; nuclear response factor, TFAM; mitochondrial transcription factor A, TNF; tumor necrosis factor, MCP-1; monocyte chemoattractant protein 1, AS160; Akt substrate 160.

Beyond ROS protection, promoting mitochondrial function is an attractive target in diabetes management, as depressed mitochondrial density and function in the skeletal muscle of type-2 diabetics has been reported [99]. In addition to stimulating the white-to-brown phenotypic switch in adipose tissue, anthocyanins also promote mitochondrial activity in skeletal muscle [100]. Oral C3G (1 mg/kg bodyweight) increased exercise performance (swimming time to exhaustion) by improving lactate and glucose metabolism via activation of PGC1-α in ICR mice. PGC1-α gene expression was increased by 3.3 and 2.4-fold in respective gastrocnemius and biceps muscles of mice receiving oral C3G compared to control. With the same comparisons, PGC1-α protein expression was increased by 2.6 and 2.8-fold. PGC1-α activation and increased mitochondrial content with C3G treatment were confirmed in C2C12 and human skeletal muscle myotube (HSMM) cultures. In the mice, blood glucose was approximately 17% higher and lactate 38% lower in C3G-treated group after exercise, demonstrating an improvement in lactate metabolism during physical activity. Increased fatty acid metabolism was also evidenced by increased gene expression of CPT1-β and significant reductions in serum ketone bodies (34%) and non-esterified fatty acids (17%) after exercise in C3G-treated mice. Similar to the aforementioned studies in adipose tissue, C3G inhibited phosphodiesterases, which increased cAMP levels. With increased cAMP, there was also an increase in intracellular calcium, which activates Ca^2+^/calmodulin-dependent protein kinase kinase (CaMKK), which phosphorylates (activates) AMPK. Stimulation of AMPK activates PGC1-α, thereby promoting mitochondrial biogenesis, evidenced by ATP generation and relevant mitochondrial gene expression in C2C12 or HSMM cell cultures. Moreover, anthocyanins can act directly as pan PPAR agonists [17,101]. Interestingly, cyanidin’s metabolite, PCA, was not shown to have these same binding or PPAR-stimulating properties [101]. Therefore, several modes exist for the modulation of mitochondrial function, regulation, and protection offered by anthocyanins. The biochemical and physiological effects of anthocyanins reviewed in the last two sections are summarized in Figure 3.

## 5. Other Considerations

### 5.1. Microbial Metabolites vs. Delivery Techniques

One criticism of anthocyanin bioactivity is its bioavailability, as it is estimated that the majority (~90–99%) of anthocyanins are not absorbed and ultimately reach the large intestine. Thus, an emergent area of investigation is the bioactivity of gut-derived metabolites of anthocyanins [84,102]. Gut metabolites of a blackberry anthocyanin extract (GMBAE) were investigated for their protective effects against cellular oxidation, ROS generation, genotoxicity, glucose metabolism, and mitochondrial function in a HepG2 model of type two diabetes [103]. Diabetic conditions were achieved via a 24-h incubation in 30 mM glucose and 0.2 mM palmitic acid before respective GMBAE treatment for an additional 24 h. The major metabolites of gut fermentation of blackberries were derived from its major anthocyanin, C3G. The total phenolic content and antioxidant activity of the fermented extract remained consistent across 48 h of fermentation. The anthocyanin profile changed dramatically across 48 h; C3G was fully metabolized by 6 h of fermentation and major phenolic metabolites included gallic acid, p-coumaric acid, 3,4-dihydroxybenzoic acid (PCA), and 2,4,6-trihydroxybenzaldehyde. Metabolically stressed HepG2s demonstrated improvements in measured outcomes after 1 and 5 µg/mL GMBAE treatments. Major findings included normalization of ROS, restoration of reduced glutathione, repolarization of the mitochondrial membrane, and up to 1.64 fold increase in HepG2 glucose consumption compared to control, which performed better than the positive control, 2 mM metformin, where glucose uptake was increased by 1.494-fold. These findings are one example that gut-derived anthocyanin metabolites also play a central role in reversing the metabolic stress of diabetes via protection of mitochondrial function. Other anti-diabetic effects of PCA and other gut-derived anthocyanin metabolites have been extensively summarized elsewhere, and they appear to modulate similar pathways as their parent compounds [104]. Studies in streptozotocin-induced diabetic rats corroborate the protective effect of PCA on mitochondrial function in both cardiac and brain tissues [105,106]. Microbial metabolites of anthocyanins appear beneficial in diabetes; the mechanistic pathways they modulate to restore euglycemia and/or prevent diabetes-induced mitochondrial dysfunction will likely gain future attention; similarly designed cellular and rodent studies, like those summarized in Section 3 and Section 4, should prioritize mechanistic and bioactive effects of microbially derived anthocyanin metabolites.

Despite the growing interest in the bioactivity of microbial metabolites of anthocyanins, other groups are determining the potential for anthocyanin encapsulation [107,108]. One group investigated the effects of 16 µM pelargonidin or nano-encapsulated pelargonidin (NP) on an L6 muscle cell model of hyperglycemia via a 2 h incubation with 1 mM alloxan [108]. Cells pretreated with pel or NP saw a rescue in proteins involved in insulin-signaling cascade (IRS1, IRS2, and PI3K) as well as GLUT4 and glucose uptake. Further, mitochondrial stress, determined by mitochondrial membrane potential, cytochrome c release, and caspase 3 and bcl2 protein expression were all improved by both anthocyanidin treatments. Notably, the NP preparation resulted in comparable or superior protection compared to nonencapsulated pel at a 10-fold less concentrated dose, indicating a more efficient delivery into the L6 muscle cells. Another microencapsulation study found up to 3-fold higher C3G absorption with a biodegradable microencapsulation technique compared to free C3G in a Caco-2 model [107]. Microencapsulation stimulated several energy-dependent forms of endocytosis (clathrin, caveolae, and macropinocytosis-related mechanisms). Anthocyanin encapsulation may offer a potential solution to longstanding challenges of achievable dose and stability of anthocyanins in humans when compared to their observed benefits in cultured cells and rodent studies. One potential concern would be losing the advantage of blocking intestinal glucose absorption via GLUT2 and sGLT1 with free anthocyanins as summarized earlier, as well as their reported inhibitory effects on carbohydrate digestion in the small intestine [104]. As novel approaches to increase anthocyanin absorption are developed, studies should determine the potential pros and cons associated with intact anthocyanins vs. microbial metabolites vs. encapsulated preparations on antidiabetic activity across intestinal, hepatic, skeletal, adipose, and other tissues thought to benefit from anthocyanins or their metabolites.

### 5.2. Beyond Glucose Transporters: How and Where Are Anthocyanins and Their Metabolites Transported in the Body?

The focus of this review was to highlight the work examining the relationship of anthocyanins with both intestinal glucose transporters and GLUT4 in peripheral tissues, and how these actions may help correct the metabolic dysfunction associated with obesity. However, it would be simplistic to not mention the complexity of absorption, digestion, metabolism, and excretion (ADME) of anthocyanins. A plethora of work into anthocyanin ADME exists, and the health effects caused by their consumption cannot be constrained to the parent compounds; both intact anthocyanin metabolites (retaining the flavonoid 3-ring structure) and their simpler phenolic acids contribute to the bioactivity observed after anthocyanin consumption. Anthocyanin fate has been described in stable-isotope human feeding studies and reports of its tissue distribution in several animal models exist. The reader is directed to a recent review exploring these topics [109]. Important considerations relevant to anthocyanin action in adipose and skeletal muscle tissues include modes of transport through the blood and entrance into adipocytes and myocytes. Relevant work has found parent anthocyanins can be transported on serum albumin, and aglycones, with reduced polarity, are capable of paracellular transport across the membrane [110,111]. Tissue distribution and the extent to which anthocyanin action relies on paracellular vs. transporter-mediated cellular entry will help explain their magnitude of effect in metabolically active tissues.

## 6. Conclusions

The significance of anthocyanin interaction with intestinal glucose transporters is that first, they interfere with glucose absorption, and second, they play a major role in anthocyanin absorption. Through the modulation of regulatory pathways including insulin signaling, adiponectin secretion, and increased AMPK, anthocyanin treatment increases both GLUT4 protein levels and translocation to the plasma membrane in several metabolically active tissues. Finally, via SIRT1, AMPK, and PGC1-α stimulation, as well as through their anti-oxidant properties, anthocyanins promote mitochondrial density and function in both skeletal muscle and adipose tissues. Through their numerous actions, dietary anthocyanins may augment nutritional approaches to alleviate obesity- and diabetes-induced metabolic dysfunction and offer protection against its reoccurrence. With an aging and expanding world population, basic and translational anthocyanin research on chronic human disease is an exemplary line of investigation within the greater subject area of plant consumption and its manifold benefits to human health. Continued practice of high-quality, well-controlled experimental research, like the studies reviewed here, will enhance our understanding of the varied ways in which a plant-based diet can optimize health. Thoroughly characterized plant bioactives, combined with the collaboration of agricultural and food scientists, can inform plant breeding programs, food processing and development to increase consumer access to functional foods and ingredients in support of healthy dietary patterns.
